# Artificial Intelligence in Thoracic Surgical Oncology: Technical Foundations and Applications in Lung and Esophageal Cancer Surgery

**DOI:** 10.3390/cancers18213438

**Published:** 2026-10-24

**Authors:** Ryan Cassidy, Sara Razzaq, Austin Howell, Miguel Leiva-Juarez, Lana Schumacher

**Affiliations:** 1 Tufts Medical Center, Boston, MA 02111, USA; 2 Boston Medical Center Brighton, Brighton, MA 02153, USA; austin.howell@bmc.org; 3 Department of Cardiothoracic Surgery, University of Pittsburgh School of Medicine, University of Pittsburgh Medical Center, Pittsburgh, PA 15213, USA

**Keywords:** artificial intelligence, thoracic surgery, robotic surgery, lung cancer, esophageal cancer, machine learning, computer vision, surgical data science

## Abstract

Background/Objectives: Artificial intelligence (AI) is increasingly being investigated in lung and esophageal cancer surgery, where treatment decisions require integrating imaging, clinical risk, and operative findings. This narrative review describes the technical foundations of surgical AI and examines its applications in thoracic surgical oncology, with particular emphasis on robotic surgery and the evidence supporting clinical use. Methods: PubMed/MEDLINE was searched for English-language publications addressing AI, machine learning, deep learning, computer vision, and robotic thoracic surgery. Targeted searches and examination of reference lists identified additional studies. Publications were selected for clinical and technical relevance, prioritizing original investigations, external or prospective validation studies, and reporting guidelines. Applications were examined across preoperative assessment, intraoperative assistance, and postoperative care. Results: Preoperative applications include pulmonary nodule characterization, prediction of nodal disease and invasive tumor features, anatomical reconstruction, and assessment of response to neoadjuvant therapy. Intraoperative systems have demonstrated anatomical segmentation, surgical-phase recognition, and augmented reality guidance. Other models address technical performance, postoperative complications, recurrence, and survival. Selected studies report improved predictive performance or earlier recognition of critical structures; however, much of the evidence remains retrospective and institution-specific. External validation is limited, and evidence that AI assistance reduces complications or improves oncologic outcomes remains insufficient. Conclusions: AI offers tools for interpreting information relevant to thoracic cancer surgery, but technical accuracy alone does not establish clinical benefit. Prospective, multicenter evaluations should determine whether AI changes consequential surgical decisions and improves patient outcomes. Reliable performance under difficult operative conditions, integration into clinical workflow, and surgeon oversight remain essential to adoption.

## Simple Summary

Surgery for lung and esophageal cancer requires balancing local control with the risks of treatment and the patient’s ability to recover. Artificial intelligence may help surgeons interpret scans, recognize critical structures during an operation, and forecast potential complications. This narrative review explains the technologies underlying these applications and examines their use throughout the perioperative environment with particular attention to robotic procedures. Although many systems show promising initial results, evidence that they meaningfully augment surgical decision making or patient outcomes remains limited. Their true clinical value will depend on reliable performance across systems and future studies should determine whether these tools help surgeons make better decisions and deliver safer, more effective cancer care.

## 1. Introduction

Lung and esophageal cancer remain among the most lethal solid organ malignancies, and for both, surgical resection remains central to curative-intent treatment. Yet thoracic surgical oncology is defined by decisions made under uncertainty. Characterizing pulmonary nodules, predicting nodal disease and invasive features, judging the appropriate extent of resection, selecting patients for and assessing response to neoadjuvant therapy, and anticipating complications such as prolonged air leak, pneumonia, and anastomotic leak. These judgments draw on heterogeneous, high-dimensional data that frequently exceed the capacity of conventional risk scores [1,2,3].

Over the past two decades, thoracic surgery has shifted decisively from open approaches toward video-assisted and robot-assisted techniques. Randomized evidence has established minimally invasive esophagectomy (MIE) as a means of reducing pulmonary complications relative to open surgery, while robot-assisted minimally invasive esophagectomy (RAMIE) has demonstrated favorable functional recovery and oncologic adequacy in resectable disease [4,5]. Parallel adoption of robotic and thoracoscopic lung resection has expanded the population undergoing complex lung cancer resections. This transition matters for artificial intelligence (AI) as much as for patients. Robotic and endoscopic platforms are not only therapeutic instruments but also dense, synchronized data sources, generating operative video, and instrument kinematics, alongside the imaging and clinical data already inherent to each case [6,7].

The maturation of these data streams has coincided with rapid advances in machine learning (ML), deep learning (DL), and computer vision. The conceptual framework of surgical data science—acquiring, annotating, and modeling data across the entire care pathway to improve interventions—has provided a unifying language for these efforts and a roadmap toward clinical translation [8,9]. In surgery broadly, AI now has demonstrated applications in the preoperative, intraoperative, and postoperative phases, encompassing risk prediction, intraoperative image analysis, and outcome forecasting [10]. Within thoracic surgery specifically, a growing body of work addresses pulmonary nodule characterization and nodal staging predictions, three-dimensional bronchovascular reconstruction and operative planning, intraoperative anatomy and surgical-phase recognition, and prediction of postoperative complications and survival. Esophageal applications increasingly target neoadjuvant response, critical-structure recognition during robotic esophagectomy, and leak prediction [3,11,12]. Despite this momentum, the field remains somewhat uneven, with much of the evidence being single-center and retrospective, and technical feasibility has too often been conflated with demonstrated clinical utility [2,3]. Realizing benefit will depend less on incremental gains in model accuracy than on integration into surgical workflow under meaningful clinician oversight [10,13].

This narrative review synthesizes the current evidence regarding AI in thoracic surgical oncology, with particular emphasis on the technical foundations of AI-enabled robotic surgery and its application to lung and esophageal cancer surgery. The review examines how AI is being incorporated across the surgical pathway, evaluates the maturity and limitations of the supporting evidence, and identifies priorities for future investigation. We ground this review on the premise that AI is most likely to advance thoracic cancer surgery through carefully designed surgeon–AI collaboration, realizing that full potential will require clinically relevant validation and evidence that these technologies improve outcomes rather than analytical performance alone.

## 2. Methods

A narrative review of the literature was performed to identify publications addressing artificial intelligence in thoracic surgical oncology, with particular emphasis on robotic surgery and applications in lung and esophageal cancer surgery. PubMed/MEDLINE was searched for English-language publications and search terms included combinations of “artificial intelligence,” “machine learning,” “deep learning,” “computer vision,” “robotic surgery,” “surgical data science,” “thoracic surgery,” “lung cancer,” “pulmonary resection,” “lobectomy,” “segmentectomy,” “esophageal cancer,” and “esophagectomy.” Targeted searches were conducted for topics including surgical-phase recognition, anatomical segmentation, three-dimensional reconstruction, augmented reality, robotic kinematics, technical-skill assessment, operative risk prediction, and postoperative complications.

Reference lists of relevant original studies, reviews, and consensus statements were examined to identify additional publications. Because the literature on thoracic surgical AI remains heterogeneous and includes both clinical studies and feasibility investigations, articles were selected according to their relevance to the scope of the review. Priority was given to original clinical studies, multicenter investigations, external or prospectively validated studies, consensus and reporting guidelines, and reviews that provided important technical or clinical context. Studies from other surgical specialties were included selectively when they described foundational technologies or concepts directly relevant to robotic thoracic surgery.

## 3. Technical Foundations of Surgical AI

Artificial intelligence is an umbrella term describing computational systems designed to perform tasks that ordinarily require human intelligence. Most contemporary applications in surgery use machine learning, in which algorithms identify patterns from data rather than relying exclusively on predefined rules. DL is a subset of machine learning that uses multilayer neural networks to learn increasingly complex representations. These distinctions are important because “AI” encompasses methods with substantially different data requirements, interpretability, and potential clinical applications [14,15].

The choice of model, therefore, is largely dependent on the clinical question and the structure of the available data. Conventional statistical and machine-learning methods, including logistic regression, random forests, gradient-boosted trees, and support-vector machines are commonly applied to structured variables such as demographics, laboratory values, comorbidities, and operative characteristics. Deep neural networks are particularly useful for high-dimensional data. Convolutional neural networks and vision transformers can analyze radiological images and operative video, while temporal models can evaluate sequences of events occurring during an operation. Natural-language processing can extract information from pathology reports, operative notes, and other clinical documents. Multimodal models seek to integrate several of these sources into a unified representation of the patient and procedure [15,16]. Surgical AI is made uniquely challenging by the diversity and temporal complexity of its data which might include combinations of preoperative imaging, electronic health records, pathology, physiological monitoring, operative video, instrument movement, and postoperative outcomes. Unlike a static diagnostic image, an operation unfolds over time and is influenced by a wide array of potential factors. Variations in operative conduct at the individual surgeon level, changes in operative field view, and anatomical variants all have potential to complicate AI-based technologies in the operating room. Consequently, the meaning of an image or movement often depends on the procedural context in which it occurs. Surgical data science provides a framework for combining these data streams and relating technical events to clinical processes and outcomes [8,9].

Developing a surgical AI system begins with defining the intended clinical use and the outcome or feature that the model is expected to identify. Data must then be collected, curated, and annotated. Ground truth may be established through pathology, postoperative outcomes, expert review, or consensus among surgeons. For video-based studies, annotation may require labeling anatomical structures, instruments, operative phases, or technical events across thousands of frames. This process is labor-intensive and may be affected by disagreement among annotators or inconsistent definitions between institutions. Standardized terminology and surgical-process models are therefore important for creating datasets that can be compared and combined [17,18].

The available data are typically divided into training, validation, and test sets. The training set is used to estimate model parameters, the validation set guides model selection and tuning, and the test set provides a final assessment on unseen data. Separation should occur at the patient level and, when appropriate, at the surgeon or institutional level. Otherwise, information shared between datasets can produce data leakage and an overly optimistic estimate of performance. Other common threats to model development include small sample sizes, missing data, class imbalance, overfitting, and shortcut learning, in which a model relies on incidental features rather than clinically meaningful information [19,20]. Model evaluation must be matched specifically to the intended task. Classification models are often reported using sensitivity, specificity, precision, recall, and the area under the receiver-operating-characteristic curve (AUC). Segmentation models commonly use the Dice coefficient or intersection over union to quantify overlap between predicted and reference regions. Predictive models should also be assessed for calibration, which measures agreement between predicted and observed risk. For real-time systems, latency, temporal stability, false-alert frequency, and performance under changing operative conditions may be as important as overall accuracy [21].

**Figure 1:**
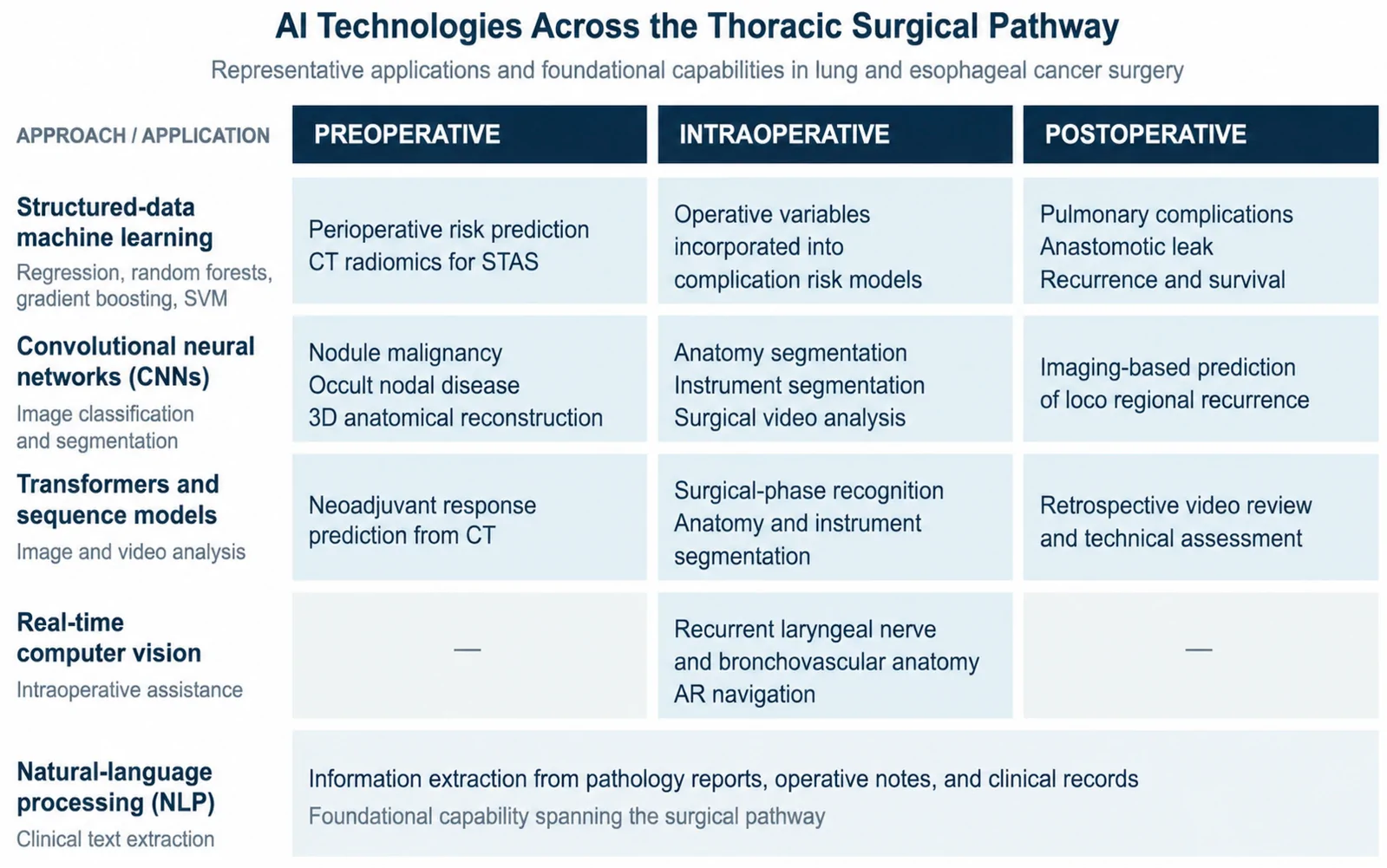
Overview of artificial intelligence approaches and representative applications across the preoperative, intraoperative, and postoperative spectrum of thoracic surgical oncology.

Internal validation of a model alone does not establish clinical utility in lieu of external validation to determine whether performance is maintained across different populations, operative platforms, and clinical contexts. Prospective evaluation must then establish whether introducing the system leads to measurable changes. These would include improvements in decision making, workflow, surgical quality, complications, oncologic outcomes, or recovery. Thus, surgical AI should be evaluated along a translational continuum of technical performance, external validity, workflow integration, and clinical impact. This distinction provides the framework for interpreting the evidence reviewed in the subsequent sections. Figure 1 illustrates how these computational approaches support different tasks across the thoracic surgical oncology pathway, linking their technical foundations to the clinical applications discussed in subsequent sections.

## 4. AI-Enabled Robotic Surgery

Robotic surgery has particular relevance to thoracic oncology because it combines minimally invasive access with a digitally controlled operative environment. Lung resection and esophagectomy require precise dissection around critical hilar and mediastinal structures. Robotic platforms enhance visualization and instrument dexterity within these confined anatomical spaces, but contemporary systems remain teleoperated as the surgeon interprets the operative field, controls the instruments, and makes all substantive decisions.

Artificial intelligence adds a computational layer to the robotic platform. Depending on its function, an AI system may analyze operative data retrospectively, recognize anatomy or procedural events in real time, assess performance, or execute a narrowly defined task. These functions exist along a continuum from passive data capture to context-aware assistance, task-level automation, and conditional autonomy [7,10,22]. As the data presented in the coming sections illustrate, the clinically relevant near-term model for thoracic surgery is not replacement of the surgeon but augmentation of perception, planning, and surgical awareness. It is also important to note that although the purpose of this section is to highlight AI systems on robotic platforms, much of the work discussed would also be feasible in traditional VATS and some of the included studies actually involve AI in traditional thorascopic cases.

In addition to high-resolution video, robotic systems can record instrument position, movement paths, camera activity, clutch use, energy activation, and system events. This information, coupled with other patient-specific data in the perioperative setting, makes robotic surgery a potentially powerful environment for surgical data science and AI development [7,10,16]. An individual instrument movement in a vacuum has little meaning; its interpretation depends on whether the surgeon is dissecting a bronchovascular structure, dissecting a lymph node, mobilizing the esophagus, or performing an anastomosis. Context-aware AI aims to decipher not only what action takes place, but where the operation is and where it is likely to go. To this end, AI models could deduce whether certain actions are appropriate in a phase-specific manner [7,16]. Development of this contextual awareness in a clinically reliable format and in a live operation remains difficult. Thoracic operations vary in terms of tumor location, patient anatomy, receipt of neoadjuvant therapies, adhesive disease from prior operations, and surgeon technique. For example, the progression of a lobectomy may change depending on the completeness of the fissure or bronchovascular anatomy while an esophagectomy takes place in multiple fields and technique varies between approaches.

Computer vision enables identification of visual features in robotic operative videos. Foundational work in other surgical subspecialties have shown that DL models can detect instruments, anatomy, and procedural phases. Sarikaya et al., for example, showed that neural networks could detect and localize surgical instruments in robot-assisted procedures [23]. Applications in thoracic surgery are particularly intriguing when one considers the difficult dissection seen, for example, in post-induction lung resection where hilar fibrosis can obscure operative planes [24]. Anatomical segmentation could in theory highlight potential danger-areas or support real-time overlays. Early studies in the thoracic surgery literature demonstrate feasibility but significant limitations remain. LungSurg, a generative AI model developed by Liang et al., combines segmentation of intrathoracic anatomy and instruments with phase classification during thoracoscopic lobectomy [25]. The clinical significance of such technologies has yet to be fully proven as anatomy identification does not necessarily signal a meaningful reduction in injuries. Recognition of operative stages has numerous potential applications within thoracic surgery. Workflow recognition can allow automated recognition of procedural phases that, in its most simple form, could assist with segmenting surgical videos for postoperative review. Earlier studies on surgical workflow segmentation showed promise in laparoscopic cases [26,27]. AI integration into robotic thoracic surgery cases has already demonstrated technical and clinical feasibility. Specifically, phase recognition has been demonstrated in robotic esophagectomy cases, and anatomy identification has been demonstrated in lung resection [28,29].

From an educational standpoint, the data-rich environment of robotic platforms provides a natural path for performance improvement. Video and kinematic instrument data can quantify instrument motion paths and provide useful insights into bimanual coordination and economy of motion. This has been demonstrated in studies examining open, laparoscopic, and robotic surgery [30,31]. Trainees early in their thoracic surgical learning curve may also benefit from these advances. Feasibility data have shown that AI and AR guided tracking and registration is possible in real-time lobectomies [28]. The clinical and educational significance of such technologies has yet to be proven, though larger scale studies may be able to evaluate this. The feasibility study by Sadeghi et al. does show one important technical feature. Lung isolation during thoracic cases does deform pulmonary anatomy, which would ordinarily make 3D overlays and structure identification difficult. However, their study using DL, AR and interactive and dynamic simulated realities shows that this can be overcome at least in a carefully selected case on the robotic platform [28].

## 5. Applications in Lung Cancer Surgery

In lung resection, AI has been studied most extensively through CT-based models that address which nodules to target and how much lung to resect. Intraoperative applications are fewer and remain at the feasibility stage, but they build directly on the computer vision and augmented reality (AR) foundations described above.

## 6. Pulmonary Nodule Characterization and Surgical Candidacy

Conventional nodule calculators such as the Brock (PanCan) model rely heavily on size, and deep learning (DL) models aim to capture morphologic features beyond it. The strongest recent evidence comes from a National Lung Screening trained algorithm that was externally tested on three European screening trials [32]. At 100% sensitivity for cancers diagnosed within 1 year, the model classified 68.1% of benign nodules as low-risk compared with 47.4% for PanCan, a 39.4% relative reduction in false-positive findings. In size-matched malignant and benign nodules, its AUC was 0.79 compared with 0.60 for PanCan. Performance is lower for incidental nodules seen in routine practice. In a multicenter incidental-nodule cohort, two DL models achieved AUCs of 0.74 and 0.72 compared with 0.63 for Brock [33]. Models can also fail on atypical cases without signaling it. When uncertainty estimation was added to one DL model, its AUC fell to 0.62 in the most uncertain nodules, compared with 0.90 to 0.93 in the remaining nodules [34].

What matters most for surgeons is whether these tools lead to meaningful clinical impacts. In a recent multireader study, AI assistance increased the proportion of malignant nodules recommended for biopsy or resection from 55% to 72%, without a significant change in invasive recommendations for benign nodules (18% vs. 17%) [35]. These data alone do not prove clinical utility across broader populations, though prospective evaluations commercial AI nodule platforms are now under way [36]. Operative decision making, especially in medically complex patients with poor physiologic reserve, can make extent of resection a major decision point. Predicted postoperative FEV1 derived from AI-based CT analysis has emerged as a potential independent predictor of pulmonary complications after lobectomy or sublobar resection, which supports its use alongside formal pulmonary function testing [37].

## 7. Extent of Lung Resection

The choice between lobectomy and sublobar resection has become a central preoperative decision in early-stage lung cancer. In randomized trials of peripheral tumors 2 cm or smaller, segmentectomy was associated with increased overall survival compared with lobectomy in JCOG0802/WJOG4607L, and sublobar resection was noninferior to lobectomy in terms of disease-free survival in CALGB/Alliance 140503 [38,39]. Both results, however, depended on careful selection: CALGB 140503 required intraoperative confirmation of node-negative disease, and locoregional recurrence was higher after segmentectomy in JCOG0802 [38,39]. Several of these selection criteria align directly with AI. Tumor size and consolidation-to-tumor ratio are currently measured by hand and leave room for inter-reader variability, and automated segmentation could standardize them. More importantly, AI has potential to help identify features that make a sublobar resection oncologically inadequate, such as occult nodal disease and spread through air spaces (STAS).

Occult nodal disease is the most studied of these features. A CT-based DL signature for occult N2 disease in clinical stage I NSCLC achieved AUCs of 0.82, 0.81, and 0.81 in internal, external, and prospective cohorts [40]. The same group’s PET/CT model was proposed as a way to guide nodal biopsy and to select patients for sublobar resection and lobe-specific nodal dissection [41]. More recently, a 3D DL model trained on 500 patients reached an AUC of 0.87 in a 400-patient external cohort and outperformed four experienced radiologists at identifying occult lymph node disease [42]. These models aim to provide preoperatively the nodal information that CALGB 140503 obtained through intraoperative frozen section, enabling an appropriate surgical plan from the outset.

STAS may partly explain the higher locoregional recurrence after sublobar resection and is therefore particularly relevant to the extent of resection. A stacking ensemble model combining CT radiomics and clinicoradiological features was developed in 965 patients and provided reliable, noninvasive, preoperative prediction of STAS in clinical stage I lung adenocarcinoma patients [43]. Pathology-based models offer a complementary approach, with AI-predicted STAS positivity associated with significantly shorter disease-free survival specifically among patients undergoing sublobar resection [44].

Many of the previous predictive models rely on the readily available preoperative CT scan. This can also be leveraged to create operative risk predictions. AI-derived predicted postoperative FEV1 was a potential independent predictor of pulmonary complications after both lobectomy and sublobar resection, which may help identify patients in whom a lung-sparing resection is preferred for physiologic rather than oncologic reasons [37].

Nearly all of these models were developed retrospectively in Asian centers, and none have been evaluated in trial populations. It is worth emphasizing these obvious limitations as accurate staging is of the utmost importance to the oncologic adequacy of surgical lung cancer management. These data remain relatively immature, and whether AI leads to improved care and outcomes across the thoracic surgical spectrum has yet to be demonstrated.

## 8. Intraoperative Localization and Anatomy Recognition

Localizing small or subsolid nodules is more difficult in robotic surgery because tactile feedback is absent. AI can be used to this end by generating 3D models that drive navigation. In a prospective feasibility study, AR-guided marker placement with a head-mounted device resulted in oncologically adequate margins for every resected nodule on frozen section [45]. Newer systems automatically generate anatomic models from non-enhanced CT in 2–3 min to guide intraoperative navigation during segmentectomy [46]. Registration of preoperative anatomy to the moving thoracoscopic image remains the main barrier to AR guidance, and the first robotic AR-assisted lobectomies relied on manual registration [28]. Our group is currently developing a markerless approach that aligns a CT-derived three-dimensional pulmonary artery model with transformer-based segmentation of the live video, without fiducial markers or tracking hardware; early work supports feasibility, but registration accuracy and overlay stability have not yet been validated.

**Figure 2:**
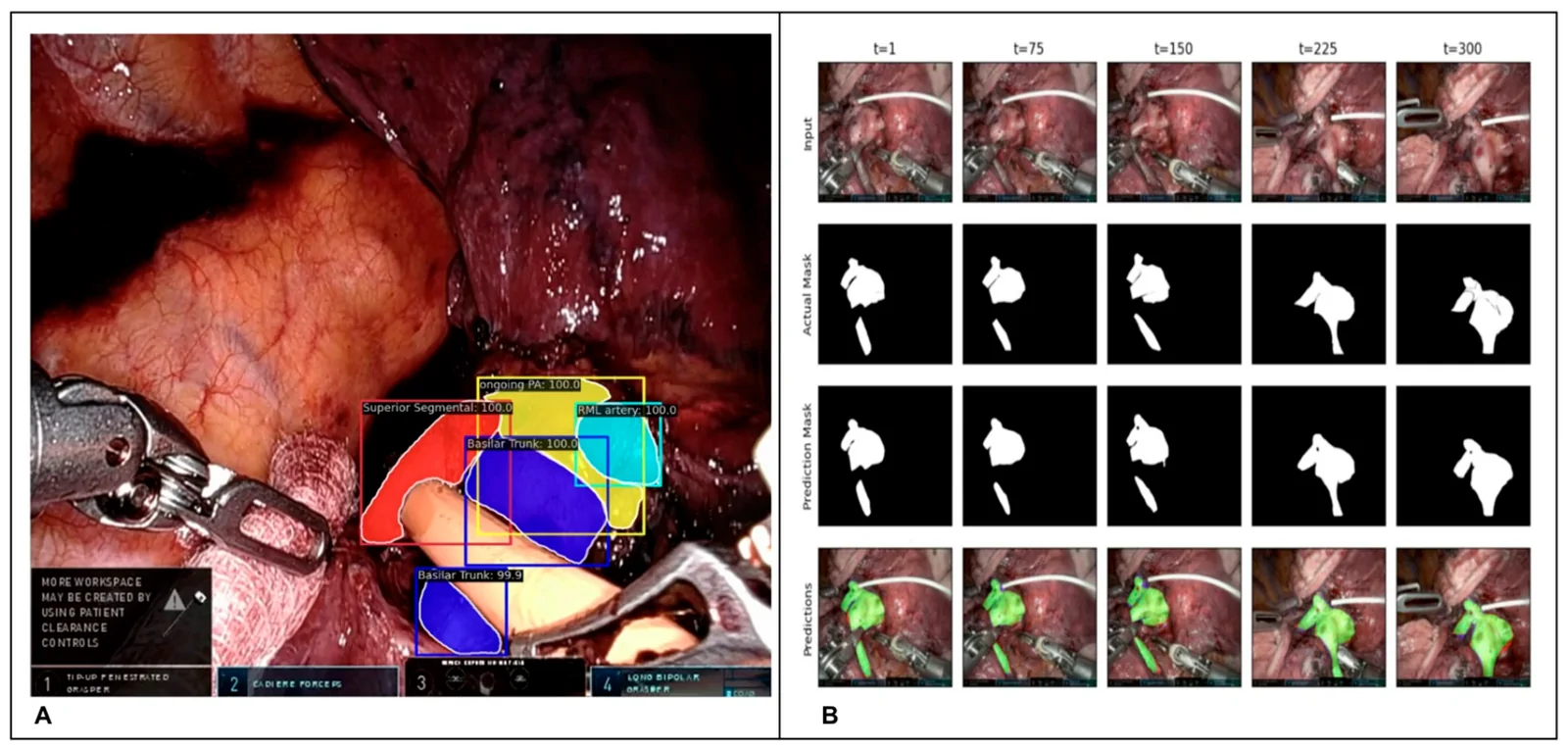
Pulmonary artery recognition during robotic lobectomy. (**A**) Multi-class segmentation identifying individual pulmonary artery branches during robotic right lower lobectomy. (**B**) Memory-augmented video segmentation (XMem) tracking the pulmonary artery across consecutive frames of a robotic right lower lobectomy; rows show the input frame, the expert-annotated ground truth, the model’s predicted mask, and the prediction overlaid on the operative field. Images from the authors’ ongoing work.

Video-based anatomy recognition extends the LungSurg and robotic AR work described earlier [25,28]. In 95 robotic right upper lobectomies, a transformer-based model segmented the pulmonary artery and vein (Dice 0.78), phrenic nerve (Dice 0.66), and bronchus (Dice 0.57), although structures were not detected consistently from frame to frame [47]. A thoracic nerve recognition system used in 10 lung cancer operations was rated satisfactory by surgeons, with almost no time lag relative to the surgical monitor [48]. Work from our group has focused on pulmonary artery recognition during robotic lobectomy, where vascular injury remains among the most feared complications. Early models based on a conditional generative adversarial network and on Mask R-CNN segmented the pulmonary artery accurately on validation data but generalized less consistently to unseen patients [49,50]. A memory-augmented video segmentation model (XMem), which draws on preceding frames to handle occlusion and camera motion, was trained on right lower lobectomies and maintained accuracy on unseen left upper lobe cases (mIoU 85.6%) and left lower lobe cases (mIoU 74.8%), although performance was lower for the inferior pulmonary vein (59.2%) [51,52] (Figure 2). These small, single-institution studies illustrate both the promise of cross-lobar transfer and the limitations when current models venture beyond their training anatomy. Clinical outcome improvement has not yet been associated with any of these technologies, which is an obvious limitation to its widespread adoption. There is also little evidence to support reliable, real-time anatomy recognition in a deflated lung. This, of course, is a necessary obstacle to overcome with future work. AI may also assess tumor features during surgery. VPI-Net identified visceral pleural invasion on thoracoscopic images with AUCs of 0.84 to 0.94, outperforming two surgeons and a radiologist, and patients with low VPI scores had a longer time to progression after sublobar resection [53].

Lymphadenectomy assessment is the least developed of these applications, even though it is a core quality measure within the field of lung cancer surgery. Current work is limited to recognizing nodes and adjacent nerves during dissection. One nerve recognition study prioritized station 4L dissection because identifying the left vagus and recurrent laryngeal nerves there is clinically important [48]. To our knowledge, no model yet verifies which nodal stations were dissected or links video-derived completeness to pathologic nodal yield, which makes this a clear target for robotic platforms that already record the necessary data.

## 9. Applications in Esophageal Cancer Surgery

Like all thoracic surgery, the diagnosis and management of esophageal disease has been greatly impacted by the recent and dramatic improvement of artificial intelligence tools. This section will discuss recent and developing artificial intelligence applications in diagnosis, surgical and non-surgical management, and overall care of the patient with esophageal disease with a particular focus on esophageal malignancy and esophagectomy.

Esophageal malignancy remains a challenging condition with a five-year survival rate of less than 25%. With cases projected to increase through 2040, new strategies for diagnosis and management of both squamous cell (ESCC) and adenocarcinoma (EAC) are needed [54]. Machine learning and artificial intelligence have shown great promise in assisting with the management of this condition in several areas. This section will review the various applications for AI in the arena of esophageal malignancy.

### 9.1. AI in the Workup of Esophageal Malignancy

Diagnosis of esophageal cancer remains challenging, with endoscopy remaining the mainstay of diagnosis. However, even in the hands of an expert, the sensitivity of white light endoscopy alone can be as low as 48% [55]. Computer-Aided Detection (CADe) systems have begun to see clinical use in colonoscopy for improved polyp detection with the GI Genius system attaining FDA approval in 2019, with one meta-analysis demonstrating a 27% relative improvement in adenoma detection across seven included RCTs [56]. With the success of such systems, investigation into similar techniques for detection of Barrett’s esophagus, esophageal dysplasia and early neoplasia is well underway. These efforts are already showing great promise, with many CNN-based systems demonstrating sensitivity and specificity >90% and often outperforming human endoscopists [57]. Also important to guiding treatment decisions is assessment of depth of tumor invasion, and work is also being performed in this sphere. One study by Nakagawa et al. created a DL algorithm capable of differentiating mucosal from superficial and deep submucosal invasive lesions with accuracy similar to human endoscopists [58]. With such promising results, clinical adoption seems likely if not inevitable, though at this point, it is important to note that current technologies have not been proven to improve outcomes. The American Society for Gastrointestinal Endoscopy (ASGE) has previously published recommendations regarding sensitivity and specificity thresholds for any new diagnostic technologies which are frequently being met by such technologies [59].

### 9.2. The Role of AI in the Operating Room

Minimally Invasive Esophagectomy (MIE) has been shown to be safe and effective when compared to an open approach leading to increased worldwide adoption [60]. As a result, increasing amounts of image and video data have become available for development and training of computer vision models intended for intra-operative use. The applications of such models are numerous but can be divided mainly into the categories of anatomic recognition and image segmentation, phase recognition, and performance review. Segmentation is a computer vision technique whereby a model is trained to divide images or videos into constituent parts. One of the most mature applications of this technology pertaining to esophagectomy is identification of the recurrent laryngeal nerve (RLN). A study by Furube et al. designed a model capable of highlighting the RLN in real time and found that its incorporation decreased time to confident detection of the RLN by a statistically significant margin [61]. Given that traction and thermal injuries to the nerve typically occur prior to identification this kind of technology has the potential to reduce such injuries by shrinking the window for their occurrence and representing an area where AI could meaningfully reduce negative outcomes. Broader models are also a target of current research, such as one published recently by Van Jaarsveld et al., which achieved an overlap score of 0.72 across 12 label classes [62]. This study is notable in particular for the large number of images included for segmentation. Hand in hand with the concept of anatomic recognition is that of surgical phase recognition. This technology attempts to divide a procedure such as esophagectomy into defined phases (for example azygos vein division or esophageal transection) and train a model to accurately identify the phase of the surgery being performed. One study by Takeuchi et al. in 2022 was able to achieve this for nine phases with an accuracy of 84% [29]. The earlier referenced study by Van Jaarsveld was able to achieve an accuracy of 82.8 percent across thirteen surgical phases with their highest-performing model.

These technologies both have tremendous potential to aid in surgical education. As discussed earlier anatomic segmentation has the potential to aid surgical trainees in rapidly identifying critical anatomy even in cases where dissection has not yet been fully completed, helping to prevent injuries to difficult-to-identify structures such as the recurrent laryngeal nerve and thoracic duct. Phase recognition can assist trainees in understanding normal workflow of esophagectomy, evaluating time spent in a given surgical phase compared to experts to track performance, and notify the surgeon when major deviations from typical phase progression have occurred [63].

### 9.3. Outcome Forecasting and Patient Selection

Diagnosis and surgical management aside, AI may have a place in esophagectomy in helping to predict both patient response to treatment and assess risk of postoperative complications. For example, a study by Zhang et al. developed a radiomics model that aimed to predict patient response to neoadjuvant immunochemotherapy (nICT) in esophageal squamous cell carcinoma (ESCC) and found that the developed Vision-Mamba model was able to demonstrate accuracy between 83-91% on both internal and external validation sets [64].

Prediction of complications may also be assisted with the use of AI tools. Various studies examining imaging characteristics, pre-operative patient features, and intra-operative data have been performed to attempt to quantify a patient’s risk for postoperative complications. A recent systematic review by Satija et al. identified nineteen studies attempting to design models capable of predicting post-operative outcomes after esophagectomy. The studies were a combination of prospective and retrospective reviews including both adenocarcinoma and ESCC. Studies examined a variety of post-operative outcomes including early recurrence, 30-day general morbidity/readmission, specific complications including anastomotic leak and pulmonary complications, and disease-free and overall survival. Results were variable but overall favorable, and the addition of histopathologic and radiomic data consistently improved the predictive accuracy of the models compared to those who used clinical data alone [65].

One uncommon but potentially devastating complication of esophagectomy is anastomotic leak. Tools that could predict the likelihood of a leak based on patient factors or visually based on the anastomosis could be monumental in reducing the morbidity of this procedure. In one study by Pollmann et al. in 2025, the researchers were able to design an unsupervised clustering network that was able to quantify the mean intensity and time to max intensity to assign patients an ICG perfusion score, which correlated with observed anastomotic leak rates [66]. This was a small, retrospective sample limiting the generalizability of these results. Randomized, prospective studies will be needed to fully assess the reliability of these models and their impact on patient outcomes.

## 10. Translation to Clinical Utility, Limitations, and Future Directions

The applications reviewed above illustrate the breadth of AI development in thoracic surgical oncology, but the transition from technical feasibility to routine clinical use requires a clearer connection between model performance and patient benefit. Accurate identification of the pulmonary artery does not establish that an overlay prevents vascular injury, just as prediction of anastomotic leak does not establish that acting on that prediction improves recovery. Each application should therefore begin with a defined clinical decision, an intended user, and an actionable output. Evaluation should establish whether providing that information improves care beyond existing practice. DECIDE-AI addresses an important stage in this transition by providing reporting guidance for early clinical evaluations, encompassing clinical performance and safety [67]. In thoracic surgery, validation should account for differences in patient populations, neoadjuvant treatment, operative approaches, and imaging or varying operative approaches and minimally invasive platforms. For video-based applications, evaluation should include difficult operative conditions such as bleeding, hilar fibrosis, and adhesive disease, rather than only clearly exposed structures. Multicenter collaboration would allow investigators to test these conditions across a broader range of surgeons and institutions. Shared definitions of anatomical labels, procedural phases, and postoperative outcomes would also improve comparability between studies. And for clinical prediction models, transparent reporting of patient selection, missing data, model development, and performance is essential to determining whether results are applicable across populations. TRIPOD+AI provides an updated reporting framework for prediction models developed using either regression or machine-learning methods [68].

Successful integration will also depend on how surgeons actually interact with AI systems. A technically accurate system may have limited value if its output arrives too late, obscures the operative field, or interrupts attention at a critical moment. Early evaluations should therefore examine the combined performance of the surgeon and the system, including whether assistance changes decisions, increases workload, or encourages inappropriate reliance on incorrect predictions. In thoracic applications, relevant questions include whether an anatomical overlay remains useful during dissection, and whether a risk estimate prompts a clearly defined change in perioperative management. User training, disagreement with model outputs, and the circumstances in which assistance is disregarded should be documented. These considerations are consistent with the emphasis on human–AI interaction in DECIDE-AI and CONSORT-AI [69]. Performance should be monitored after implementation, particularly when software, equipment, or clinical practice changes. Evaluation across patient subgroups and institutions is necessary to identify unequal performance that may be concealed by aggregate results. The FUTURE-AI consensus guideline addresses these broader requirements through recommendations spanning the entire process of an AI model, from development and testing through implementation [70].

A practical research priority, relative to thoracic surgery, is to connect preoperative imaging and clinical risk with intraoperative events and postoperative outcomes. Such integration could help investigators determine when operative information adds value beyond data already available before surgery. Increasing model complexity, however, should be justified by an improvement in a defined clinical task. The most useful next studies will establish where AI assistance changes management, which patients benefit, and what resources implementation requires. Progress toward routine use will depend on reproducible clinical benefits and a clearly defined role for surgeon oversight.

## 11. Conclusions

A central challenge in thoracic surgical oncology is deciding how to achieve local control without exceeding the reserve of a patient. AI will earn a place in this work if it helps surgeons connect information across the care pathway relating preoperative risk to operative findings, recognizing when a planned dissection or resection requires reconsideration, and anticipating complications early enough to intervene. The studies reviewed here establish promising technical capabilities. However, clinical value of those capabilities remains incompletely characterized throughout the literature. Our responsibility as surgeons is to shape their development around consequential decisions and to test whether their use improves care. The measure of progress will be whether AI helps surgeons operate with better judgment and achieve better and more durable clinical and oncologic outcomes.

## Data Availability

No new data were created or analyzed in this study. Data sharing is not applicable to this article.
